# A Careful Insight into DDI-Type Receptor Layers on the Way to Improvement of Click-Biology-Based Immunosensors

**DOI:** 10.3390/bios14030136

**Published:** 2024-03-06

**Authors:** Sylwia Karoń, Marcin Drozd, Elżbieta Malinowska

**Affiliations:** 1 Department of Medical Diagnostics, Centre for Advanced Materials and Technologies CEZAMAT, Warsaw University of Technology, Poleczki 19, 02-822 Warsaw, Poland; sylwia.karon.dokt@pw.edu.pl; 2 Chair of Medical Biotechnology, Faculty of Chemistry, Warsaw University of Technology, Noakowskiego 3, 00-664 Warsaw, Poland

**Keywords:** DNA-directed immobilization, DNA-protein conjugates, self-assembled monolayers, surface plasmon resonance, conjugates purification, receptor layer formation

## Abstract

Protein-based microarrays are important tools for high-throughput medical diagnostics, offering versatile platforms for multiplex immunodetection. However, challenges arise in protein microarrays due to the heterogeneous nature of proteins and, thus, differences in their immobilization conditions. This article advocates DNA-directed immobilization (DDI) as a solution, emphasizing its rapid and cost-effective fabrication of biosensing platforms. Thiolated single-stranded DNA and its analogues, such as ZNA^®^ and PNA probes, were used to immobilize model proteins (*anti*-CRP antibodies and SARS-CoV nucleoprotein). The study explores factors influencing DDI-based immunosensor performance, including the purity of protein-DNA conjugates and the stability of their duplexes with DNA and analogues. It also provides insight into backfilling agent type and probe surface density. The research reveals that single-component monolayers lack protection against protein adsorption, while mixing the probes with long-chain ligands may hinder DNA-protein conjugate anchoring. Conventional DNA probes offer slightly higher surface density, while ZNA^®^ probes exhibit better binding efficiency. Despite no enhanced stability in different ionic strength media, the cost-effectiveness of DNA probes led to their preference. The findings contribute to advancing microarray technology, paving the way for new generations of DDI-based multiplex platforms for rapid and robust diagnostics.

## 1. Introduction

Microarrays, or multi-analyte assays, have served as efficient tools for high-throughput diagnostics, involving the miniaturization of various assays on a single substrate. They find applications in genetic diagnostics (DNA/RNA arrays) as well as proteomics and immunodiagnostics (protein/immunoarrays) [1,2,3]. Nonetheless, the potential of protein microarrays, as opposed to DNA arrays, is still constrained by a number of technological obstacles. While DNA is a relatively stable oligomer with well-defined physicochemical characteristics, practically independent of its sequence, proteins refer to a heterogeneous group of biomolecules that are generally more fragile than DNA [4]. As a result, selecting an optimal immobilization approach that assures protein availability and precise surface orientation is critical. While nucleic acids are commercially accessible with various chemical modifications, allowing for appropriate surface orientation, proteins are more diverse, making it challenging to develop immobilization methods with broad applicability [5].

Nowadays, it is highly desirable to design microarrays that enable numerous measurements in a row with a detection method that can be performed on a routine basis. Such an approach is possible when regeneration is conducted without harming the layer, or when it is possible to remove receptors and rapidly reconstruct the entire receptor layer [6,7,8]. The ability of complementary oligonucleotide strands to hybridize has been introduced into more complex assemblies, such as protein microarrays obtained via DNA-directed immobilization (DDI). This implementation enables miniaturized, rapid, low-cost, efficient, and site-specific fabrication of biosensing platforms. DDI requires conjugating antibodies with complementary DNA strands to those immobilized on the surface [8]. This functionalization has been employed in label-based [5,9] and label-free [10,11] immunoplatforms.

Directional immobilization of proteins through DNA anchors has been reported in the development of biosensors [12,13], i.e., for profiling of extracellular vesicles [5], detection of hormones [14], circulating cancer biomarkers [15], and viral pathogens [11]. DDI also found applications in protein arrays, as well as in platforms for multiplex kinetic analysis and cell organization [16]. Even though constructing a functional receptor layer in a multiplex format for the analysis of protein samples is much more challenging, several emerging problems are often overlooked in DDI. These include selecting an appropriate anti-fouling agent, optimizing the probe surface density, and purifying the conjugates. The attachment of DNA anchors to protein receptors can be realized by covalent methods and biological affinity [17]. Conjugating the two biomolecules is also quite troublesome to control, regardless of the strategy employed. Nevertheless, conjugate valency (DNA to protein ratio) aspects are also underestimated in constructing DDI-based bioreceptor platforms [4,18].

It should also be noted that negatively charged DNA chains tend to repel each other, resulting in inefficient hybridization, and problems with controlling the surface density of bioreceptors. Using DNA analogues instead of conventional DNA may improve detection accuracy because it can discern a perfect match from a single base-pair-mismatched strand. The interaction of the analogues and DNA is unaffected by the ionic strength of the buffer, and they can hybridize with the corresponding DNA in a low ionic strength environment in the absence of divalent cations [19]. We expect that the properties of new generations of nucleic acid analogues may still have undiscovered advantages for applications in the development of receptor layers via DDI.

In this work, we have thoroughly investigated the importance of the selected factors, such as the quality and purity of DNA-protein conjugates, the composition of the oligonucleotide monolayer, and the type and surface density of anchoring probes for the improvement of the quality of receptor layers for potential applications in label-free, DDI-based immunosensing. For this purpose, thiolated single-stranded DNA and its analogues—ZNA^®^ and PNA probes (sequences of 15 or 48 nt.)—were examined as oligonucleotide probes for the immobilization of two model proteins. Mouse *anti*-CRP antibody and nucleocapsid protein of SARS-CoV were conjugated with ssDNA *ligand strand* sequence of 48 nucleotides, then purified through ion chromatography, and used for the formation of a regenerable DNA-based layer for detection of viral inflammation biomarkers. Based on the results of SPR and SPRi measurements, the influence of purity and stoichiometry of the conjugates on their capability for DDI-based immobilization was investigated. Additionally, the differences in the hybridization kinetics for the conjugates and free DNA and the influence of the type of oligonucleotide probes on hybridization efficiency were examined. The selection of the backfilling agent was carried out to provide the best receptor layer affinity and binding specificity. The relationship between the oligonucleotide probe charge and the protein-DNA conjugates’ binding efficiency and stability was also determined. The findings presented in this article explore aspects relevant to the molecular recognition process at interphase for the further improvement of DDI-based immunoplatforms.

## 2. Materials and Methods

### 2.1. Reagents and Materials

Gold SPR slides (50 nm gold layer thickness) were purchased from Horiba Scientific (Palaiseau, France) (SPR imaging studies) and BioNavis Ltd. (Tampere, Finland) (multi-parametric SPR kinetic studies). DNA and ZNA^®^ oligonucleotide sequences were purchased from Metabion GmbH (Planegg, Germany). A thiolated *PNA probe* was purchased from Panagene Co., Ltd. (Daejeon, Republic of Korea). The sequences used in this study were as follows:

- *ligand strand* (48 nt.) 5′-NH_2_-C_6_-ATC AGT ACT TGT CAA CAC GAG CAG CCC GTA TAT TCT CCT ACA GCA CTA-3′
- *DNA probe (long)* (48 nt.) 5′-SH-C_6_-TAG TGC TGT AGG AGA ATA TAC GGG CTG CTC GTG TTG ACA AGT ACT GAT-3′
- *DNA probe* (15 nt.) 5′-SH-C_6_-TAG TGC TGT AGG AGA-3′
- *PNA probe* (15 nt.) 5′-SH-C_6_-TAG TGC TGT AGG AGA-3′
- *ZNA^®^ probe* (15 nt.) 5′-SH-C_6_-TAG TGC TGT AGG AGA-(spermine)_3_-3′

Additionally, 6-mercaptohexanol, poly(ethylene glycol) methyl ether thiol (average M_n_ = 800 Da) (referred to as *PEG-800*), polyclonal goat *anti*-mouse secondary antibody, polyclonal rabbit IgG antibody, sodium chloride, potassium chloride, sodium hydrogen phosphate, sodium dihydrogen phosphate and potassium dihydrogen phosphate were from Sigma-Merck (Poznań, Poland). Ammonia (25%), hydrogen peroxide (30%), sulfuric acid (96%), hydrochloric acid (35–37%), glycine and sodium hydroxide were from Chempur (Piekary Śląskie, Poland). *ɑ*-hydroxy-*ω*-mercapto PEG (average M_n_ = 3.0 kDa) (referred to as *PEG-3k*) was from Rapp Polymere GmbH (Tübingen, Germany). Monoclonal mouse *anti*-hCRP antibody, clone line 6405 was from Medix Biochemica (Espoo, Finland). SARS-CoV Nucleocapsid recombinant protein expressed in *Escherichia coli* was from Thermo Fisher Scientific (Warsaw, Poland). proFIRE^®^ Amine Coupling Kit for proteins (>5 kDa) was from Dynamic Biosensors GmbH (Munich, Germany). All solutions were prepared using deionized (DI) water (conductivity < 0.055 μS/cm at 20 °C, TOC < 1.0 ppb).

### 2.2. Surface Preparation of SPR Gold Slides and Ex Situ Immobilization of Oligonucleotide Probes

SPR gold-coated slides were rinsed with DI water and then cleaned using 15 min immersion in “basic piranha” solution (25% ammonia, 30% hydrogen peroxide, and DI water in volumetric ratios 1:1:3) at 70 °C. Next, slides were rinsed with deionized water, dried under compressed air, and soaked in “acidic piranha” solution (3:1 (*v*/*v*) mixture of conc. sulfuric acid and perhydrol) for 1 min. Subsequently, slides were rinsed with DI water, dried in a compressed air stream, and cleaned under UV/ozone (Ossila Ltd., Sheffield, UK) for 30 min. DNA, PNA, or ZNA^®^ oligo probes were diluted to 0.5 µM solutions in phosphate-buffered saline (PBS) pH = 7.2 (unless stated otherwise). Such solutions (or mixtures with a backfilling agent—typically *PEG-800*—at a molar ratio of 1:4) were dispensed on a clean Au SPR slide by manual spotting (0.5–8 µL per spot) and incubated for 30 min. The whole Au surface was rinsed with DI water and covered with the backfilling agent—typically 50 μM aqueous solution of *PEG-800* for 20 min.

### 2.3. Conjugation of Receptor Proteins with ssDNA Anchors and Conjugates Purification

The conjugation process was carried out with amine-terminated *ligand strand* DNA oligonucleotide (3 nmol) and 200 µg of the protein: *anti*-hCRP antibody (M_w_ ~ 130 kDa, 1.54 nmol) or SARS-CoV nucleoprotein (M_w_ ~ 62.5 kDa, 3.2 nmol) using the proFIRE^®^ Amine Coupling Kit, according to the developed conjugation protocol. In the first step, the *ligand strand* sequence was incubated for 20 min at room temperature with a molar excess of the crosslinker in a 500 mM Na_2_HPO_4_/NaH_2_PO_4_ buffer, pH 7.2, with 1.5 M NaCl. After removing the excess of the crosslinker by using Zeba^TM^ spin desalting columns 7 K MWCO, the NHS ester-modified oligonucleotide was incubated overnight with 200 μg of the selected protein at 4 °C. Then, the protein-oligonucleotide conjugate was purified via preparative ionic chromatography using a proFIRE^®^ instrument (Dynamic Biosensors, Munich, Germany). Separation was carried out by the gradient elution method in 50 mM phosphate buffer pH 7.2 with a linear increase of NaCl concentration (from 0.15 M to 1.5 M). After chromatographic purification, 12 fractions (with a volume of ~ 700 μL each) were collected. The concentration of DNA conjugates in each fraction was evaluated by a built-in optical detector operating at a fixed wavelength of 260 nm. In addition, the conjugates and their components were qualitatively characterized based on absorption spectra recorded in the UV range (200–340 nm) using Lambda25 spectrophotometer (PerkinElmer, Waltham, MA, USA) equipped with quartz cuvettes with an optical path length of 1 cm.

### 2.4. Ex Situ Immobilization of Protein Conjugates by DNA-Directed Immobilization (DDI)

To immobilize protein-DNA conjugates, SPR slides modified with a selected type of oligonucleotide probe were used according to the protocol described in Section 2.2. Chromatographically purified fractions of the DNA-protein conjugates (in a concentration of ~100 nM in 50 mM phosphate buffer pH 7.2 with 150 mM NaCl) were dispensed as spots of 0.5 μL on the DNA-covered gold chip. After 1 h incubation, slides were rinsed with PBS and dried under compressed air.

### 2.5. Multi-Parametric SPR Measurements and Kinetic Analysis

Kinetic studies on the immobilization of thiolated oligonucleotide probes and their interactions with *ligand strand* target DNA and DNA-protein conjugates were carried out with an MP-SPR Navi™ 220A NAALI device equipped with an autosampler (BioNavis Ltd., Tampere, Finland). PBS pH 7.4 was employed as a running buffer at a 20 μL/min flow rate. Immobilization of the thiolated oligonucleotide probes and surface blocking was performed directly in a microfluidic system (both injections lasted 10 min at a flow rate of 20 μL/min), using the same reagents as for ex situ immobilization. PBS buffer pH 7.4 was injected into the reference channel as a negative control instead of conjugate. Consecutive injections of increasing concentrations of complementary DNA (*ligand strand*) and its conjugate with *anti*-hCRP antibody were carried out to determine the hybridization kinetics. In the case of the *ligand strand* DNA, the upper concentration level was 10.82 μM (dilution factor = 5), while for the DNA-*anti*-hCRP conjugate, the upper concentration level was 12.52 nM (dilution factor = 2). 100 mM sodium hydroxide (20 μL/min, 4 min) was used for regeneration between target injections. The kinetic evaluation of the complementary *ligand strand* DNA and *anti*-hCRP conjugates interaction with the *DNA probe* was performed with TraceDrawer^®^ software, v 1.8 (Ridgeview Instruments AB, Uppsala, Sweden) and the SPR responses were fitted according to “one to one” kinetic model.

### 2.6. Surface Plasmon Resonance Imaging Measurements

The gold SPR slide covered with ex situ immobilized DNA/PNA/ZNA^®^ oligonucleotide probes and blocked with a chosen backfilling agent was inserted into the SPRi Lab Plus instrument (Horiba, France). PBS buffer with 0.05% Tween20^®^ (PBST) was employed as a running buffer. The injection of *ligand strand* (2.7 μM in PBST buffer) or the conjugate of DNA with *anti*-CRP antibody (90 nM in 50 mM phosphate buffer pH 7.2 with 150 mM NaCl) enabled real-time monitoring and comparison of different probes’ suitability for the DNA-directed immobilization via oligonucleotide duplexes formation (manual injections, flowrate = 80 μL/min, ~5 min). In turn, the injections of unconjugated, polyclonal rabbit *IgG* antibody (10 µg/mL in PBST buffer) were carried out for studies on non-specific protein adsorption. Additionally, 100 mM sodium hydroxide was used for the regeneration between injections (80 μL/min, 4 min).

### 2.7. Surface ζ-Potential Measurements of Oligonucleotide Receptor Layers

Surface ζ-potential (SZP) measurements of Au substrates and receptor layers composed of self-assembled oligonucleotide probes (DNA, PNA, ZNA^®^) were carried out employing an indirect approach using a Zetasizer Nano ZS instrument (Malvern Panalytical Ltd., Malvern, UK), according to the manufacturer’s protocol [20]. Depending on the expected surface charge, a solution of Malvern transfer standard DTS 1235 (10x diluted) in 10 mM phosphate buffer (pH 7.4) or a diluted solution of fabric softener Lenor^®^ (also known as Downy^®^) in 10 mM phosphate buffer pH 7.4 was used as an anionic/cationic tracer, respectively. SPR slide fragments were mechanically cut to 5 × 4 nm and used as substrates for SZP measurements. After cleaning and immobilization of various oligonucleotide probes (as in Section 2.2), the substrates were glued to the table between the electrodes of the measurement cell using cyanoacrylate glue. A series of measurements of the tracer’s ζ-potential at increasing distances from the examined layer (in the range of 125–750 µm) enabled the calculation of the ζ-potential directly at the interphase. The calculations were based on the linear fitting and Equation (1) [21]:

SZP = tracer ζ-potential − intercept (d = 0 µm)(1)

## 3. Results and Discussion

The development of functional and reproducible bioreceptor layers for molecular interaction studies using “click-biology” methodologies (such as DNA-directed immobilization) requires preserving possibly unaffected bioreceptor activity and efficient attachment of DNA anchor tags. The concept of “click biology”—by analogy with the more widely known term “click chemistry”—represents the convenience obtained by effortlessly attaching two moieties through bio-molecular interactions. It often refers to biotransformation processes, DNA ligation, or formation of highly ordered structures via hybridization [22]. Preferably, each bioreceptor molecule is expected to be linked to one molecule of anchor DNA, and the conjugates should be separated from the unbound components. Meeting all the prerequisites allows efficient loading of the transducer’s surface binding sites with protein-DNA conjugates. The problem of the quality of DNA-protein conjugates (as well as other receptor layers based on immobilization of tagged receptors) is often overlooked in protocols developed to date, which can negatively affect the analytical performance of as-constructed biosensors. In the case of inadequately purified conjugates, uncontrolled dilution of protein receptors may occur due to the competition of tagged proteins and free tags (e.g., DNA anchors) for binding sites. It negatively affects the sensitivity. In addition to diluting receptor layers by blunt DNA anchors, the presence of multiple-tagged receptors with disturbed stoichiometry (DNA tag to protein ratio >> 1) is also unfavorable. A multiple-tagged protein—especially in analyte-binding regions—may exhibit altered analyte binding capacity. Therefore, it is vital to control the quality of DNA-labeled receptors at the synthesis stage and to carry out the post-synthetic preparative purification of DNA-protein conjugates. The goal is to maximize the fraction of DNA-tagged proteins characterized by a 1:1 DNA-to-protein stoichiometry.

### 3.1. Purification of DNA-Protein Conjugates and SPR-Based Quality Control for DDI 

Amine coupling with the use of various NHS-based linkers, such as DBCO-NHS and maleimide-NHS, is a robust and well-characterized method of covalent conjugation of DNA with proteins [23,24]. Such conjugates are typically characterized by chromatographic methods and gel electrophoresis [25,26,27]. To provide efficient separation of DNA-protein conjugates, an already established protocol for preparative separation by ion chromatography has been employed. Variation in the retention times of DNA and other components occurs due to differences in the relative charge of the separated biomolecules. The strongly anionic character of DNA allowed for lengthened retention times of their conjugates with proteins on the ion exchange column with respect to native proteins.

**Figure 1:**
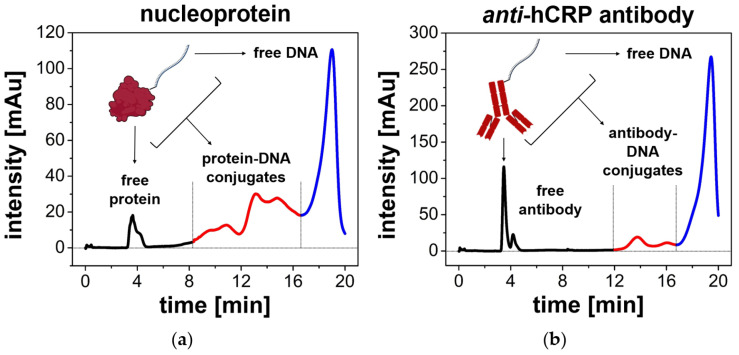
Chromatograms showing the results of separation of DNA-SARS-CoV nucleoprotein conjugates (**a**) and DNA-*anti*-hCRP antibody conjugates (**b**). The colors indicate fractions of free protein (black line), DNA-protein conjugates (red line), and free DNA (blue line). The chromatogram was captured using a spectrophotometric detector at λ = 260 nm.

In our study, pre-activated, 48-oligonucleotide-long NHS ester-terminated DNA anchors (*ligand strand* sequence) were conjugated with proteins. As model protein receptors were taken: (i) *anti*-hCRP monoclonal antibody (chosen for detection of CRP as inflammatory biomarker) and (ii) SARS-CoV nucleoprotein (receptor for serological biomarkers of COVID-19). The amine coupling reaction mixtures were subjected to preparative chromatographic separation using gradient elution and representative chromatograms are shown in Figure 1. As can be seen, non-conjugated proteins are characterized by the shortest retention times. The attachment of a single DNA strand (1:1-type conjugates) and more (multiple-tagged conjugates) resulted in a significant interaction enhancement, resulting in gradually longer retention times. Unbound DNA leaves the column as the last, as it exhibits the most anionic character. In both cases, shown in Figure 1, DNA-protein conjugates were found in the post-reaction mixture. To confirm the correct assignment of the observed signals to the respective fractions, chromatograms from the analysis of a sample containing only DNA and a mixture of protein and DNA (unconjugated) are shown in Figure S1a. In addition, the effect of NHS-based crosslinker on DNA ligand strand was examined to rule out the possibility of its spontaneous crosslinking (Figure S1b). A more in-depth discussion on the selectivity of the amine coupling and reactivity of NHS esters with exocyclic amine groups of nucleobases and the differences in coupling efficiency for individual proteins can also be found in Supplementary Material.

Similarly, analysis of subtle differences in the UV absorption spectra allowed us to observe differences in the compositions of the main chromatographic fractions. The normalized absorption spectra of the free DNA *ligand strand*, the free antibody, and the two fractions of their conjugates are shown in Figure S2, and the detailed discussion on applicability of this method for quality control of DNA-protein conjugates was provided in Supplementary Material [24,28].

**Figure 2:**
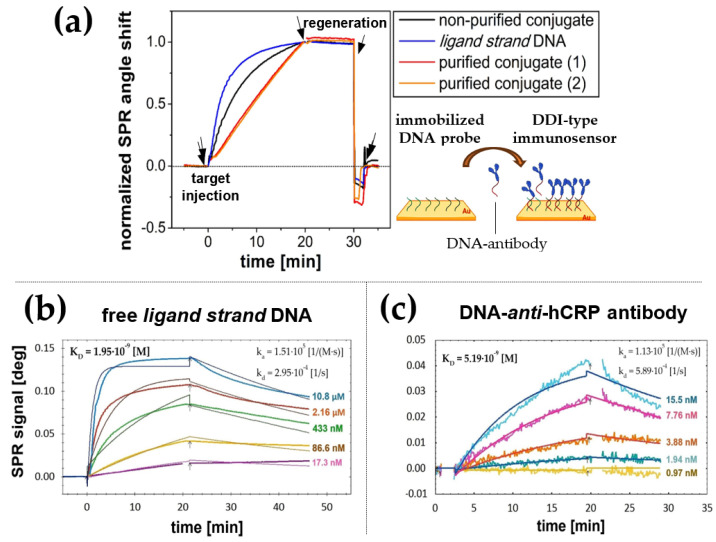
SPR responses representing the binding of targets: free DNA and DNA-antibody conjugates at different purification levels (2 fractions of purified conjugates and a non-purified reaction mixture), by an immobilized *DNA probe* (**a**). SPR responses to injections of a series of *ligand strand* DNA (**b**) and DNA-*anti*-CRP antibody conjugate (**c**) against the immobilized *DNA probe*. Colored curves represent experimental results, and black curves illustrate the fitting according to the 1:1 kinetic model.

The relevance of the purification process of DNA-protein conjugates was evaluated in our study by comparing their interaction kinetics with *DNA probes* immobilized on SPR slide (Figure 2a). Normalized sensograms allow a comparison of the binding kinetics of two fractions of DNA-mAb *anti*-hCRP conjugates (with 1:1 stoichiometry and multiply tagged) to a layer of immobilized *DNA probes*. SPR responses were also recorded for the reaction mixture after conjugation but without chromatographic purification and for free *ligand strand* sequence (corresponding to DNA tag) as a reference. As shown in Figure 2a, according to our expectations, the significant difference in the SPR response profile for the conjugates before and after purification indicates a substantial contribution of binding unconjugated DNA strands to the receptor layer. The flat, nearly linear evolution of the SPR signal at the step of conjugate association indicates a noticeably slower kinetics of its binding to the surface, typical for bulky ligands whose binding is further limited by diffusion processes [28]. The apparent difference in the association curves confirms that the hybridization of unconjugated *ligand strand* DNA is additionally favored kinetically due to lower steric constraints. 

To further analyze the binding kinetics of free DNA ligands and purified conjugates, association and dissociation rates (k_a_, k_d_) and equilibrium dissociation constant (K_D_) were further determined, as shown in Figure 2b,c. K_D_ levels of several nM indicate the high affinity of both components to immobilized DNA probes, which will promote their effective binding to the surface at low concentrations. The lower K_D_ value, which characterizes the binding of *DNA probes* to the free *ligand strand* sequence (1.95 nM) compared to binding of DNA-*anti*-CRP antibody conjugates (5.91 nM), confirms the thermodynamic preference of binding free ligands over their protein conjugates. The observed, more than 3-fold, difference in K_D_ values will translate into lower durability of probe DNA complexes with DNA-protein conjugates when compared to regular DNA–DNA duplexes. The detailed parameters of the kinetic model employed as well as the obtained results are summarized in Table S1. Residuals between fitted curves and experimental data for interactions of DNA probe with free ligand strand DNA and DNA-*anti*-CRP antibody conjugate were shown in Figure S3. Given both the relatively low rate of DNA conversion during amine coupling with proteins and the proven preference for binding non-conjugated DNA, ensuring adequate purity of DNA-protein conjugates appears particularly important. Such an approach should be reflected in an improvement of the surface density of protein receptors during DNA-directed immobilization.

### 3.2. Development of the Oligonucleotide Probe Layer Composition for DDI

#### 3.2.1. Selection of the Oligonucleotide Anchor Type

The formation of oligonucleotide duplexes through DNA-directed immobilization is based on strong and spontaneous Watson–Crick base pair interactions. Nevertheless, a specific activation barrier exists involving electrostatic repulsion of phosphate backbones of complementary DNA strands [29]. This phenomenon negatively affects both the hybridization kinetics and the thermodynamic stability of duplexes of immobilized probes and DNA-protein conjugates. Also, at an earlier stage, electrostatic repulsion may impair self-assembly of thiolated probes on Au substrates. It can result in a reduced effective surface density of protein receptors immobilized by DDI. To overcome the mutual repulsion of DNA strands, buffers with high ionic strength are typically required. These provide screening of the negative charge by the cations present in solution [30]. However, this approach imposes limitations with respect to the composition of immobilization and hybridization media. Maintaining a high ionic strength can also be troublesome in real-world applications, as it results in the risk of dissociation of DDI-immobilized receptors during washing steps.

**Figure 3:**
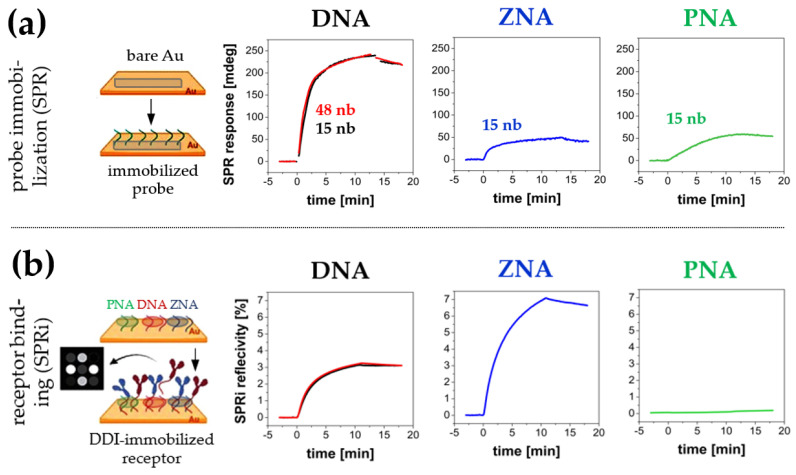
(**a**) Comparison of SPR responses, which reflect direct chemisorption of thiol-terminated DNA, ZNA^®^ and PNA probes on the surface of a gold SPR transducer during probes immobilization. (**b**) SPRi responses representing hybridization of the oligonucleotide probes with fully complementary *ligand strand* sequence. In both cases, red sensograms refer to a “long” *DNA probe* (48 nb) and black sensograms refer to a “short” *DNA probe* (15 nb).

With this in mind, we analyzed the possibility of using DNA analogues as immobilized DDI probes. To attenuate the anionic character of oligonucleotide probes, two DNA analogues of the same sequence (15 nb length) were examined: thiol-terminated peptide nucleic acid (PNA-SH) and heterobifunctional DNA with ω-mercaptohexyl and spermine trimer at both terminals, known as zip nucleic acid (ZNA^®^) [31,32]. With the replacement of DNA with PNA and ZNA^®^ analogues, the polyanionic nature of receptors is expected to be attenuated, which should facilitate the binding of complementary sequences and the persistence of duplexes. As shown in Figure 3a, DNA analogues (ZNA^®^ and PNA) had significantly lower immobilization efficiency than regular DNA. The surface density of ZNA^®^ and PNA reached 17% and 19% of the density observed for the *DNA probe*, respectively [33,34]. 

A plausible explanation for the observed phenomena lies in the complexity of the interaction mechanism of the probes with the gold surface. The surface density and spatial orientation of probes (especially for relatively long oligonucleotide sequences) are also significantly affected by direct interactions of side chains with the gold surface [35]. Both PNA, rich in electron-donor groups capable of interacting with gold atoms, and DNA labelled with oligoamine tag (ZNA^®^), promote multidentate tethering. Such a phenomenon results in the horizontal alignment of oligonucleotide strands on the gold surface, thus limiting the maximum achievable surface density and capability of subsequent hybridization. Literature reports suggest that PNA chemisorption is a two-step process and a high probe concentration in the immobilization medium is needed to obtain the desired conformation [33]. Given the reported problems in PNA SAMs formation, as well as their generally high cost and the inconsistency of preparation protocols (some recommend the addition of trifluoroacetic acid and/or organic solvent to improve PNA solubility [34]), we concluded that they were not competitive with classical DNA probes and did not continue further optimization.

In the case of ZNA^®^, relatively low surface density did not adversely affect the hybridization efficiency in PBS buffer observed by means of SPRi—on the contrary, as can be seen in Figure 3b, the obtained response of the ZNA^®^ receptor layer was nearly twice as high as that observed for the DNA layer. It can be explained by lowering the thermodynamic barrier for the *ligand strand* binding reaction due to the attenuation of electrostatic repulsion of DNA strands at the initial stage of duplex formation. High hybridization efficiency at low surface density makes the *ZNA^®^ probe* a good candidate as an alternative to DNA as high-binding anchors for DNA-directed immobilization. In contrast, the formation of PNA–DNA duplexes was not observed under the examined conditions.

In parallel with the study of DNA analogues, we also verified the impact of the DNA oligonucleotide probe (regular—15 nb vs. long—48 nb) on its ability to self-assemble on gold. As shown in Figure 3a (left graph), both injected sequences (*DNA probe* and *DNA probe (long)*, respectively) underwent spontaneous chemisorption. The similar values of the resonant angle shifts (236 mdeg and 239 mdeg, respectively) at the end of the association stage reflect the similar surface density of the associated mass. However, given the significant difference in molecular weight of regular and long *DNA probes* (mass ratio 1.0: 3.05), the observed values of SPR angle shift indicate an approximately threefold higher (expressed in molar terms) surface density of short *DNA probes* [36]. Given the observed lack of a noticeable difference in the binding capacity of the *ligand strand* sequence (Figure 3b—left graph), probes with a length of 15 nb were used during further studies. Such probes proved equally effective as the full-length complementary sequences for capturing oligonucleotide-tagged proteins in DDI. 

**Figure 4:**
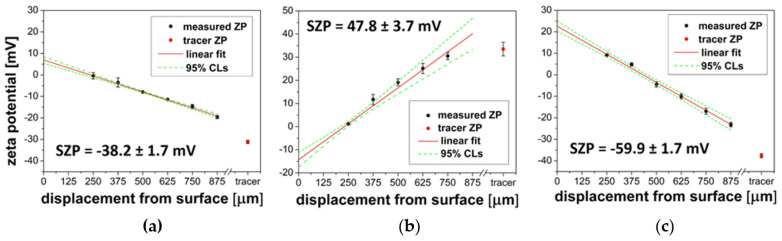
Plots of measured ζ-potential of tracer particles against displacement from the surface of the following: (**a**) Au@DNA probe, (**b**) Au@ZNA^®^ probe, (**c**) bare Au. Red dots represent native ζ-potential of the tracer (undisturbed by the measured surface). Extrapolated ZP values for d = 0 were used to indirect determination of surface ζ-potential.

To evaluate the influence of the type of oligonucleotide probe on its surface charge, values of surface ζ-potential of Au substrates before and after modification with *DNA* and *ZNA^®^ probes* were determined (Figure 4). As can be seen, the introduction of a terminal polycationic chain into the structure of DNA dramatically alters its surface charge. At near neutral pH (7.4), both bare gold (*via* anions coordinatively bound to the surface) [37], as well as Au modified with *DNA probe*, as expected, are characterized by an anionic surface (represented by a negative surface ζ-potential (ZP), −59.9 mV and −38.2 mV, respectively). In contrast, for ZNA^®^, the measured ZP value was +47.8 mV. It can be expected that these significant differences in the character of the surface can affect the stability of oligonucleotide duplexes employed in DDI. The surface charge can also affect non-specific interactions of sample components with immobilized probes [38,39].

**Figure 5:**
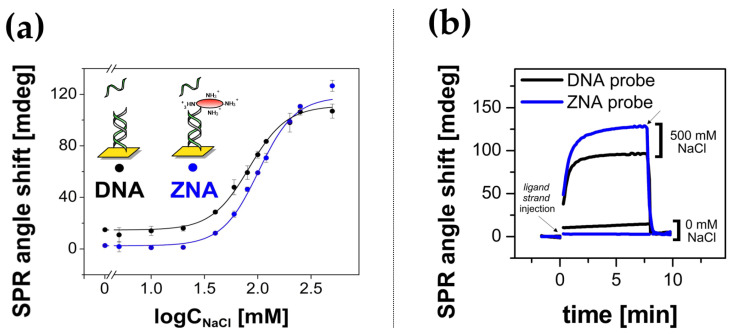
(**a**) The relationship between *ligand strand* target concentration and its hybridization efficiency with immobilized DNA (black dots) and *ZNA^®^ probes* (blue dots). Relative signal values were calculated from differential SPR sensograms after the association equilibrium was reached (*n* = 2), (**b**) exemplary SPR responses recorded for *ligand strand* injections in high ionic strength medium (10 mM phosphate buffer pH 7.4 with 500 mM NaCl) and low ionic strength medium (10 mM phosphate buffer pH 7.4 with 0 mM NaCl). Distilled water was used as the running buffer.

The stabilities of DNA–DNA and ZNA^®^–DNA duplexes were compared by SPR studies in a medium with low ionic strength. For this purpose, a series of equimolar *ligand strand* sequence injections in buffer with varying Na^+^ concentration (regulated by the concentration of NaCl in phosphate buffer) were carried out. Examples of recorded SPR responses and SPR signal values as a function of Na^+^ concentration for *DNA* and *ZNA^®^ probes* are shown in Figure 5. Contrary to our expectations, beyond the previously observed higher efficiency of duplex formation in PBST buffer, no other advantages of the ZNA^®^ receptor layer over the DNA layer were observed.

Notably, no enhanced efficiency of ZNA^®^–DNA duplex formation was observed in low ionic strength media, as evidenced by the similar hybridization efficiency profiles shown in Figure 5a. Likewise, both types of hybrids showed the typical DNA tendency to rapid, water-induced cleavage of the double helix, as can be seen in the sensograms provided in Figure 5b. Given the lack of significant advantages of *ZNA^®^ probes*, it was therefore decided to prefer cheap and readily available *DNA probes* for anchoring DNA-protein conjugates in the framework of further studies. A summary of the applicability of DNA and its analogues as probes for DDI can be found in Table S2. It lists their immobilization and hybridization efficiencies and the benefits of their use.

#### 3.2.2. Selection of Non-Receptor Components

Another aspect determining the application potential of Au substrates with immobilized oligonucleotide probes in DDI-based biosensor design is their resistance to non-specific adsorption of proteins. The origin of such interferences can be both protein conjugates and components of matrices of real samples. For DDI-based biosensors, hybridization via DNA tags should be the only effective way to anchor protein receptors. Therefore, it is necessary to minimize passive adsorption and surface fouling by other proteins. The appropriately designed receptor layer gives a lower background response and ensures comfortable and efficient regeneration under controlled conditions.

**Figure 6:**
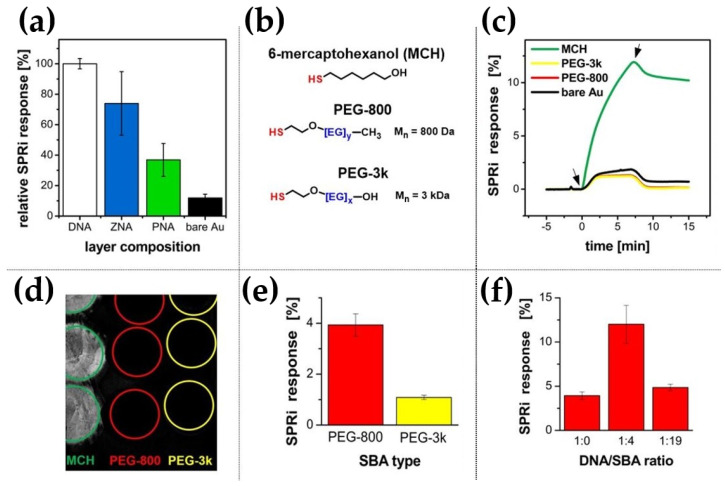
(**a**) Normalized SPRi signals recorded as responses of DNA, PNA, and ZNA^®^ monolayers and bare gold to injection of unconjugated rabbit IgG antibody (10 µg/mL), (**b**) structures of thiolated backfilling agents (BA) used within this study, (**c**) SPRi sensograms (left), (**d**) SPRi differential image depicting the interactions of monolayers composed of backfilling agents (MCH, *PEG-800*, *PEG-3k*) and bare gold with unconjugated rabbit IgG antibody (10 µg/mL), (**e**) and (**f**) SPRi signal values, which represent hybridization efficiencies of mixed monolayers containing immobilized *DNA probe* and *ligand strand* sequence, as a function of (**e**) type of PEG-based backfilling agent used (molar ratio of *DNA probe* to BA = 1:4), (**f**) molar ratio of DNA probe to *PEG-800* in the immobilization mixture (*n* = 3).

As shown in Figure 6a, single-component monolayers composed only of oligonucleotide probes do not protect against the adsorption of native rabbit IgG antibodies chosen as a model interferent. It is evidenced by a significant increase of SPRi signals, indicating fouling of DNA, ZNA^®^, and PNA monolayers under measurement conditions suitable for DDI-based sensors. Remarkably, the surface charge of the layer does not significantly affect the adsorption process as both *DNA* and *ZNA^®^ probes* are susceptible to protein fouling. In contrast, bare gold was characterized by several times lower IgG adsorption (~10% of the value observed for *DNA probe*—see Figure 6a). The results suggest that electrostatic interactions do not play a dominant role in the adsorption of proteins on oligonucleotide-modified gold surfaces.

A careful selection of backfilling agents was carried out to ensure better assembly of oligonucleotide probes on the Au substrate. The resulting mixed monolayer is expected to be characterized by an increased ability to capture DNA *ligand strand* together with the improvement of anti-fouling properties of the obtained receptor layer [38,39,40]. To choose the optimal backfilling agent, three thiol-terminated polar ligands differing in size and structural flexibility were compared: 6-mercaptohexanol, oligo (methyl ether thiol) with a molecular weight of 800 Da (*PEG-800*), and thiolated PEG with a molecular weight of 3 kDa (*PEG-3k*) (Figure 6b). SPRi studies of the interactions between native rabbit IgG antibody and monolayers of individual backfilling agents showed that the tested components have different anti-fouling properties. The effectiveness of the blocking agent increases dramatically with the introduction of flexible and highly hydrophilic polyether segments into its structure, which can be easily observed in the case of *PEG-800* and *PEG-3k*, (sensograms in Figure 6c and SPR image in Figure 6d). PEG derivatives are very effective in suppressing the adsorption of a model protein interferent, in contrast to 6-mercaptohexanol, which is typically employed as a component of DNA biosensors.

Covering the surface of a biosensor with a bulky surface-blocking agent carries the risk of creating a steric hindrance to surface processes. Shielding of oligonucleotide probes by long-chain PEG ligands can hinder the anchoring of DNA-protein conjugates during the DDI process. Therefore, the effect of both PEG-type backfilling agents (in the form of a mixed layer with *DNA probes*) on *ligand strand* sequence binding capacity is shown in Figure 6e. The significant, nearly 4-fold, decrease in hybridization efficiency for the *PEG-3k*-containing mixed monolayer compared to the analogous *PEG-800*-containing layer confirms the significance of the above-postulated steric effect on the efficiency of DNA-directed immobilization. Therefore, employing *PEG-800* as a component of the receptor layer for immobilizing DNA-protein conjugates appears to be a reasonable compromise that combines receptor binding efficiency and good anti-fouling properties. 

Further, the effect of the surface density of *DNA probes* on the binding efficiency of the complementary sequence was also tested. For this purpose, *DNA probes* were co-immobilized with thiolated *PEG-800* as a dilution agent at different molar ratios. It was shown that the “diluted” layer obtained by co-adsorption of *DNA probe* with *PEG-800* at a molar ratio of 1:4 showed more than 120% better binding efficiency than an analogous layer formed by two-step immobilization. In such an approach, surface blocking followed the incubation of the Au substrate with an undiluted *DNA probe*. This effect was observed over a wide range of DNA to *PEG-800* molar ratios. Finally, at a 19-fold excess of the blocking agent, the layer reached a similar binding capacity as the layer fabricated with an undiluted receptor (Figure 6f). At first glance, the surprising result indicates the significant influence of spatial freedom on solid-phase hybridization capacity. As we expect, this effect will be further enhanced when more bulky ligands in the form of DNA-tagged protein receptors are employed. Presumably, dense DNA layers, through their high charge concentration and insufficient freedom to adopt a double-helix conformation, are not conducive to efficient hybridization. For this reason, mixed-type layers (*DNA probe* to *PEG-800* molar ratio = 1:4) were used to further study DNA-based layers.

However, it should be emphasized that the optimal surface density (expressed as the ratio of DNA probe to PEG-800) may vary depending on the length and structure of the specific oligonucleotide probe (including the length of the aliphatic linker or the presence of non-binding spacer). Therefore, given the possibility of individual design of DNA anchor sequences depending on the application, we have given up more detailed optimization in this field. In turn, for *ZNA^®^ probes* (as molecules of partially neutralized global charge and characterized by a relatively low probe surface density—see Figure 3a), backfilling was scheduled as a separate step after the self-assembly of undiluted *ZNA^®^ probes* on the Au surface.

**Figure 7:**
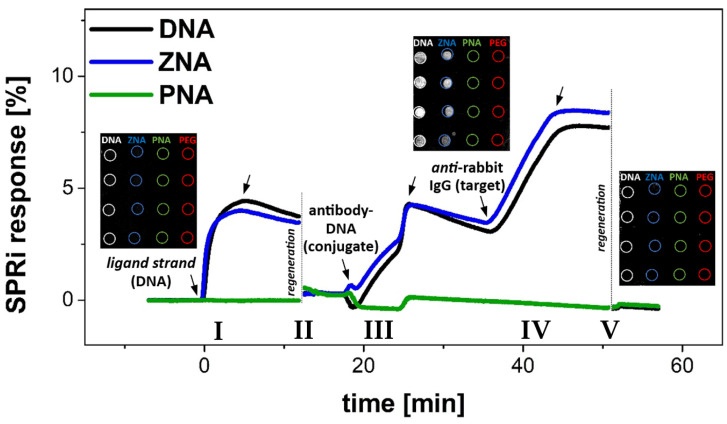
Average values of SPRi signal changes for the optimized composition of DNA-modified layer (co-immobilization of *DNA probe* and *PEG-800* at molar ratio 1:4, (0.1 µM *DNA probe* + 0.4 µM *PEG-800*), PNA- and ZNA^®^-modified layer (immobilization of *PNA* and *ZNA^®^ probe* followed by surface blocking with *PEG-800*). The sensograms show the subsequent processes: (I) binding of unconjugated *ligand strand* sequence (preliminary quality control of the immobilized probes), (II) surface regeneration by means of injection of 50 mM NaOH, (III) injection of DNA-*anti*-hCRP antibody conjugate, (IV) injection of *anti*-mouse IgG (as model analyte), (V) complete regeneration of DDI-based biosensor. PBST buffer pH 7.4 was used as a running buffer and for all dilutions. Insets show differential SPRi images, which visualize the observed differences in reflectivity for different oligonucleotide probes.

To demonstrate that DNA and ZNA^®^ anchoring layers with a previously optimized composition (mixed layers of oligonucleotide probes and *PEG-800*) provide good quality substrates for DDI, three injections of the components of the receptor layers and the model target were carried out sequentially. As shown in Figure 7, consecutive injections of unconjugated *ligand strand* (500 nM), mouse IgG antibody-DNA conjugate (40 nM—based on DNA concentration), and *anti*-mouse IgG (10 µg/mL) resulted in sensor responses consistent with those expected from previous experiments. The ability to hybridize with free DNA ligands and their conjugates was demonstrated by immobilized *DNA* and *ZNA^®^ probes*. The first provided a slightly higher surface density of hybridized *ligand strand* represented by the observed reflectivity shifts (3.49% for DNA vs. 3.25% for ZNA^®^), while for DNA-antibody conjugates the binding efficiency was slightly higher for *ZNA^®^ probes* (6.14% for ZNA^®^ vs. 5.48% for DNA). In both cases, the immobilized antibodies showed the capacity for immunoreaction with *anti*-mouse IgG antibodies used as model analytes. The analyte binding efficiency (from a sample containing the target antibody at a concentration of 1 µg/mL) was readily observable in real time using SPRi (see inset in Figure 7).

The observed reflectance changes for both types of layers were very similar (4.6% for DNA and 4.7% for ZNA^®^) with a complete lack of response for the PNA layer (for which, however, no oligonucleotide and protein receptors bound to the surface after immobilization were detected). It indicates a significant correlation between the surface density of receptors immobilized via DDI and the efficiency of immunoreaction. These conclusions are similar to the findings of Simon et al. for studies of the formation of PNA–DNA and DNA–DNA duplexes in monolayers [41]. 

Equally important from the point of view of the reusability of DDI biosensor substrates is the observed excellent regeneration efficiency. Injection of 50 mM NaOH—responsible for the breakdown of the double helix—at the same time ensures very efficient removal of immunocomplexes of antibody-DNA conjugates with the captured secondary antibodies. The signal recovery to the baseline level, i.e., before immobilization of *DNA/ZNA^®^ probes*, is observed after a single regeneration cycle (SPR signal increase does not exceed 0.2% for each of the tested oligonucleotide probe), as shown in Figure 7. It confirms good anti-fouling properties of the developed DDI platforms as well as their stability under immobilization, analyte binding, and regeneration conditions. Notably, the results of a parallel experiment carried out using SARS-CoV nucleoprotein-DNA conjugates and anti-nucleoprotein mAb turned out to be very similar. This observation points to the major advantage of the developed strategy, which is its versatility—the immobilization occurs in a similar manner and conditions, even for receptor proteins of significantly different structures and properties. 

The appropriate design of the anchor layer has been shown to provide both efficient “click-biology-based” assembly of receptors on oligonucleotide platforms and enable their interaction with model analytes. Ultimately, matrix-type platforms with receptor proteins of different origin (such as recombinant antigens or monoclonal antibodies) are intended to be used for the construction of multiplex biosensors. They can operate both in a label-free format and as highly sensitive platforms employing additional optical labelling, which is currently the subject of our research studies. Such biosensors can find application in detection of various targets, e.g., viral disease biomarkers (i.e., specific antibodies or biomarker-like antigens) in real samples.

## 4. Conclusions

DNA-directed immobilization as a strategy for receptor layer assembly has proven to have many advantages over traditional, covalent approaches. It employs only hydrophobic and/or ionic interactions between the biomolecules efficient and reversible coupling. DNA anchors allowed for rapid layer regeneration and re-immobilization without using additional reactants. The immobilization reaction occurs in mild conditions (pH about 7.0, regardless of the pI of the protein). DNA as the anchoring molecule is relatively cheap, readily available, stable, and can be stored for a long time without risk of degradation. The only disadvantages are the need for protein-DNA conjugation, careful and time-consuming conjugate purification, and relatively low coupling efficiency, leading to protein loss at the conjugation stage. We have thoroughly investigated the impact of a number of aspects important to the fabrication of protein biosensors through DDI. The importance of the synergistic effect of factors such as the quality and purity of DNA-protein conjugates, the composition of the oligonucleotide monolayer, and the type and surface density of anchoring probes has received little awareness so far, which may have resulted in suboptimal analytical performance of DDI-based biosensors. Our study demonstrated key differences in the kinetics and thermodynamics of hybridization between unconjugated DNA and its conjugates with *anti-*CRP antibodies and SARS-CoV nucleoprotein. The lower K_D_ value (1.95 nM) of *ligand strand* binding than that of the conjugates (5.91 nM) confirms the thermodynamic preference for binding free ligands over their protein conjugates. A significantly lower binding rate and increased protein-DNA conjugate volume have been shown to alter its reactivity and binding specificity in hybridization reactions with immobilized, complementary probes. It was reflected in the use of anchor layers with low density of oligonucleotide probes and appropriate backfilling agents with improved anti-fouling properties (preferably based on PEG derivatives). Next, we compared three types of thiolated oligonucleotide anchors (based on regular DNA and their analogues: PNA and ZNA^®^) for the construction of DDI platforms. We have chosen standard DNA anchors as the most cost-effective and virtually inferior to their ZNA^®^ analogues regarding the facility and efficiency of immobilization on gold and stability of DDI duplexes with protein receptors. Mixed monolayers composed of DNA and ZNA^®^ with an optimized composition have proven to be high-binding and regenerable platforms for constructing immunosensors through a DDI strategy. The appropriate surface density and retained high affinity of the DDI-immobilized receptors allows for the label-free immunodetection of protein analytes of diagnostic significance, while also providing excellent regenerability and reusability of the platforms. We believe that the proposed improvements in DDI technology will enable its widespread implementation in the construction of versatile protein arrays based on platforms routinely employed in conventional DNA arrays. 

## Data Availability

The data presented in this study are available on request from the corresponding author.

## Supplementary Materials

The following supporting information can be downloaded at https://www.mdpi.com/article/10.3390/bios14030136/s1, Figure S1: (a) Chromatograms showing the results of separation of unconjugated SARS-CoV nucleoprotein (solid black line) and the mixture of unconjugated SARS-CoV nucleoprotein with ligand strand DNA (dashed red line). The arrows indicate fractions of free protein (retention time around 3.5 min) and unconjugated DNA (retention time around 18 min). (b) Chromatograms of ligand strand sequence before (solid black line) and after treatment with NHS-based crosslinker (dashed red line) in the absence of protein. Chromatograms were captured using a spectrophotometric detector at λ = 260 nm [26,27,42,43,44,45]; Figure S2: Normalized absorption spectra of selected fractions of post-conjugation mixture as a result of DNA-anti-hCRP antibody conjugate separation; Figure S3: Residuals between fitted curves and experimental data for interactions of *DNA probe* with fre-e *ligand strand* (a) and DNA-*anti-*CRP antibody conjugate (b); Table S1: Detailed statistics data characterizing the used kinetics binding model; Table S2: A comparison of the different types of oligonucleotide probes examined within this study.

## References

[1] Aparna G.M., Tetala K.K.R. (2023). Recent Progress in Development and Application of DNA, Protein, Peptide, Glycan, Antibody, and Aptamer Microarrays. Biomolecules.

[2] Li S., Song G., Bai Y., Song N., Zhao J., Liu J., Hu C. (2021). Applications of Protein Microarrays in Biomarker Discovery for Autoimmune Diseases. Front. Immunol..

[3] Walter J., Eludin Z., Drabovich A.P. (2023). Redefining Serological Diagnostics with Immunoaffinity Proteomics. Clin. Proteom..

[4] Watson E.E., Winssinger N. (2022). Synthesis of Protein-Oligonucleotide Conjugates. Biomolecules.

[5] Brambilla D., Sola L., Chiari M. (2021). Advantageous Antibody Microarray Fabrication through DNA-Directed Immobilization: A Step toward Use of Extracellular Vesicles in Diagnostics. Talanta.

[6] Langer A., Hampel P.A., Kaiser W., Knezevic J., Welte T., Villa V., Maruyama M., Svejda M., Jähner S., Fischer F. (2013). Protein Analysis by Time-Resolved Measurements with an Electro-Switchable DNA Chip. Nat. Commun..

[7] Cléry A., Sohier T.J.M., Welte T., Langer A., Allain F.H.T. (2017). SwitchSENSE: A New Technology to Study Protein-RNA Interactions. Methods.

[8] Washburn A.L., Gomez J., Bailey R.C. (2011). DNA-Encoding to Improve Performance and Allow Parallel Evaluation of the Binding Characteristics of Multiple Antibodies in a Surface-Bound Immunoassay Format. Anal. Chem..

[9] Huang S., Wang W., Li J., Zhang T., Liang Y., Wang Q., Jiang Z. (2021). Multifunctional DNA Mediated Spatially Confined Assembly for Antibody Orientation: Surpassing Sensitivity and Accuracy for Rituximab Detection. Chem. Eng. J..

[10] Ünlü N.L., Kanik F.E., Seymour E., Connor J.H., Ünlü M.S. (2017). DNA-Directed Antibody Immobilization. Biosensors and Biodetection.

[11] Seymour E., Daaboul G.G., Zhang X., Scherr S.M., Ünlü N.L., Connor J.H., Ünlü M.S. (2015). DNA-Directed Antibody Immobilization for Enhanced Detection of Single Viral Pathogens. Anal. Chem..

[12] Bernardinelli G., Oloketuyi S., Werner S.F., Mazzega E., Högberg B., de Marco A. (2020). A Compact Nanobody-DNAzyme Conjugate Enables Antigen Detection and Signal Amplification. New Biotechnol..

[13] Al-Amin R.A., Muthelo P.M., Abdurakhmanov E., Vincke C., Amin S.P., Muyldermans S., Danielson U.H., Landegren U. (2022). Sensitive Protein Detection Using Site-Specifically Oligonucleotide-Conjugated Nanobodies. Anal. Chem..

[14] Ladd J., Taylor A.D., Piliarik M., Homola J., Jiang S. (2008). Hybrid Surface Platform for the Simultaneous Detection of Proteins and DNAs Using a Surface Plasmon Resonance Imaging Sensor. Anal. Chem..

[15] Ambrosetti E., Paoletti P., Bosco A., Parisse P., Scaini D., Tagliabue E., De Marco A., Casalis L. (2017). Quantification of Circulating Cancer Biomarkers via Sensitive Topographic Measurements on Single Binder Nanoarrays. ACS Omega.

[16] Leroy L., Bombera R., Engel E., Calemczuk R., Laplatine L., Baganizi D.-D.R., Marche P.N., Roupioz Y., Livache T. (2014). Photothermal Effect for Localized Desorption of Primary Lymphocytes Arrayed on an Antibody/DNA-Based Biochip. Lab Chip.

[17] Dovgan I., Koniev O., Kolodych S., Wagner A. (2019). Antibody-Oligonucleotide Conjugates as Therapeutic, Imaging, and Detection Agents. Bioconjug. Chem..

[18] Yan X., Zhang H., Wang Z., Peng H., Tao J., Li X.-F., Chris Le X. (2018). Quantitative Synthesis of Protein-DNA Conjugates with 1:1 Stoichiometry. Chem. Commun..

[19] Simon L., Lautner G., Gyurcsányi R.E. (2015). Reliable Microspotting Methodology for Peptide-Nucleic Acid Layers with High Hybridization Efficiency on Gold SPR Imaging Chips. Anal. Methods.

[20] Nobbmann U. Measure Surface Zeta Potential. https://www.materials-talks.com/surface-zeta-potential-what-it-is-and-how-to-measure-it/.

[21] Corbett J.C.W., McNeil-Watson F., Jack R.O., Howarth M. (2012). Measuring Surface Zeta Potential Using Phase Analysis Light Scattering in a Simple Dip Cell Arrangement. Colloids Surf. A Physicochem. Eng. Asp..

[22] Rodríguez D.F., Moglie Y., Ramírez-Sarmiento C.A., Singh S.K., Dua K., Zacconi F.C. (2022). Bio-Click Chemistry: A Bridge between Biocatalysis and Click Chemistry. RSC Adv..

[23] El-Sagheer A.H., Brown T. (2012). Click Nucleic Acid Ligation: Applications in Biology and Nanotechnology. Acc. Chem. Res..

[24] Wiener J., Kokotek D., Rosowski S., Lickert H., Meier M. (2020). Preparation of Single- and Double-Oligonucleotide Antibody Conjugates and Their Application for Protein Analytics. Sci. Rep..

[25] Heerwig A., Kick A., Sommerfeld P., Eimermacher S., Hartung F., Laube M., Fischer D., Pietzsch H.-J., Pietzsch J., Löser R. (2023). The Impact of N^ε^-Acryloyllysine Piperazides on the Conformational Dynamics of Transglutaminase 2. Int. J. Mol. Sci..

[26] Frato K.E., Schleif R.F. (2009). A DNA-Assisted Binding Assay for Weak Protein-Protein Interactions. J. Mol. Biol..

[27] Tang Y., Cain P., Anguiano V., Shih J.J., Chai Q., Feng Y. (2021). Impact of IgG Subclass on Molecular Properties of Monoclonal Antibodies. MAbs.

[28] Marquart A., Kuncova-Kallio J., Albers M., Bombera R., Stahlberg R. (2019). Handbook of Multi-Parametric Surface Plasmon Resonance for Molecular Interaction Analysis—Theory and Practice.

[29] Gong P., Levicky R. (2008). DNA Surface Hybridization Regimes. Proc. Natl. Acad. Sci. USA.

[30] Bielec K., Kowalski A., Bubak G., Witkowska Nery E., Hołyst R. (2022). Ion Complexation Explains Orders of Magnitude Changes in the Equilibrium Constant of Biochemical Reactions in Buffers Crowded by Nonionic Compounds. J. Phys. Chem. Lett..

[31] Nothisen M., Perche-Létuvée P., Behr J.-P., Remy J.-S., Kotera M. (2018). Cationic Oligospermine-Oligonucleotide Conjugates Provide Carrier-Free Splice Switching in Monolayer Cells and Spheroids. Mol. Ther. Nucleic Acids.

[32] Zhu B., Travas-Sejdic J. (2018). PNA versus DNA in Electrochemical Gene Sensing Based on Conducting Polymers: Study of Charge and Surface Blocking Effects on the Sensor Signal. Analyst.

[33] Briones C., Mateo-Marti E., Gómez-Navarro C., Parro V., Román E., Martín-Gago J.A. (2004). Ordered Self-Assembled Monolayers of Peptide Nucleic Acids with DNA Recognition Capability. Phys. Rev. Lett..

[34] Del Bene A., D’Aniello A., Tomassi S., Merlino F., Mazzarella V., Russo R., Chambery A., Cosconati S., Di Maro S., Messere A. (2023). Ultrasound-Assisted Peptide Nucleic Acids Synthesis (US-PNAS). Ultrason. Sonochem..

[35] Drozd M., Pietrzak M.D., Malinowska E. (2018). SPRi-Based Biosensing Platforms for Detection of Specific DNA Sequences Using Thiolate and Dithiocarbamate Assemblies. Front. Chem..

[36] Sarcina L., Torsi L., Picca R.A., Manoli K., Macchia E. (2020). Assessment of Gold Bio-Functionalization for Wide-Interface Biosensing Platforms. Sensors.

[37] Li Z., Ruiz V.G., Kanduč M., Dzubiella J. (2020). Ion-Specific Adsorption on Bare Gold (Au) Nanoparticles in Aqueous Solutions: Double-Layer Structure and Surface Potentials. Langmuir.

[38] Saito N., Matsuda T. (1998). Protein Adsorption on Self-Assembled Monolayers with Water-Soluble Non-Ionic Oligomers Using Quartz-Crystal Microbalance. Mater. Sci. Eng. C.

[39] Szymczyk A., Soliwodzka K., Moskal M., Różanowski K., Ziółkowski R. (2022). Further Insight into the Possible Influence of Electrode Blocking Agents on the Stem-Loop Based Electrochemical DNA Sensor Parameters. Sens. Actuators B Chem..

[40] Havens A., El-Shaer E., Garcia L., Godino J.L., Thompson R.S. (2023). Protein Adsorption on Mixed Self-Assembled Monolayers: Influence of Chain Length and Terminal Group. Langmuir.

[41] Simon L., Gyurcsányi R.E. (2019). Multiplexed Assessment of the Surface Density of DNA Probes on DNA Microarrays by Surface Plasmon Resonance Imaging. Anal. Chim. Acta.

[42] Voigt N.V., Tørring T., Rotaru A., Jacobsen M.F., Ravnsbæk J.B., Subramani R., Mamdouh W., Kjems J., Mokhir A., Besenbacher F. (2010). Single-Molecule Chemical Reactions on DNA Origami. Nat. Nanotechnol..

[43] Gothelf K.V. (2017). Chemical Modifications and Reactions in DNA Nanostructures. MRS Bull..

[44] Verdolino V., Cammi R., Munk B.H., Schlegel H.B. (2008). Calculation of PKa Values of Nucleobases and the Guanine Oxidation Products Guanidinohydantoin and Spiroiminodihydantoin Using Density Functional Theory and a Polarizable Continuum Model. J. Phys. Chem. B.

[45] Bryantsev V.S., Diallo M.S., Goddard III W.A. (2007). PKa Calculations of Aliphatic Amines, Diamines, and Aminoamides via Density Functional Theory with a Poisson-Boltzmann Continuum Solvent Model. J. Phys. Chem. A.

