# Supplementary material for: A Careful Insight into DDI-Type Receptor Layers on the Way to Improvement of Click-Biology-Based Immunosensors
Source: Biosensors (Basel). 2024 Mar 6;14(3):136. doi: 10.3390/bios14030136 (PMC10968432; doi:10.3390/bios14030136)
Supplement: Supplementary file 1 [file biosensors-14-00136-s001.zip › biosensors-2833532-supplementary.pdf]

Supplementary Figures

Figure S1

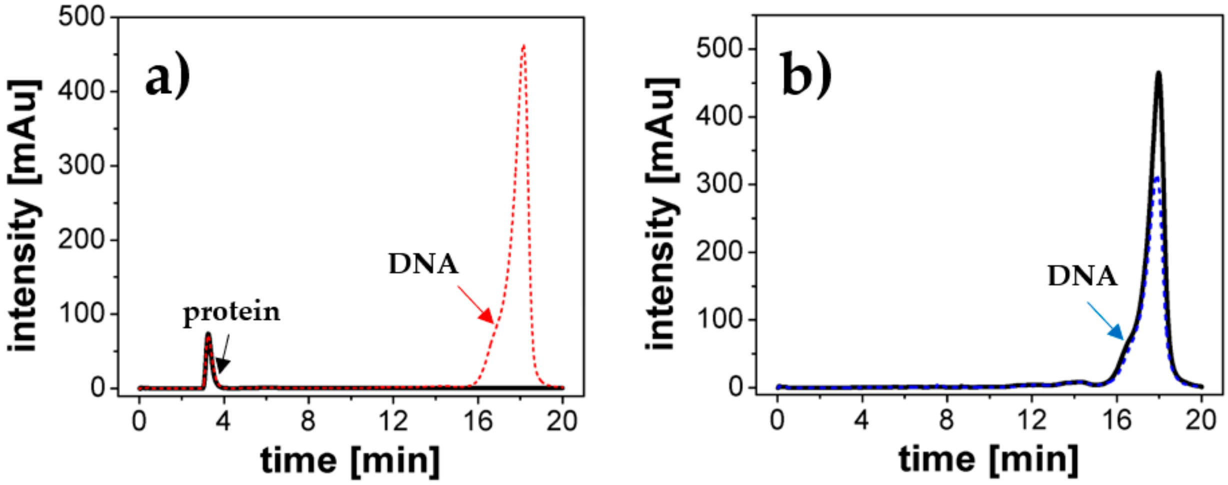

Figure S1. a) Chromatograms showing the results of separation of unconjugated SARS CoV-2 nucleoprotein (solid black line) and the mixture of unconjugated SARS CoV-2 nucleoprotein with *ligand strand* DNA (dashed red line). The arrows indicate fractions of free protein (retention time around 3.5 min) and unconjugated DNA (retention time around 18 min). b) Chromatograms of *ligand strand* sequence before (solid black line) and after treatment with NHS-based crosslinker (dashed red line) in the absence of protein. Chromatograms were captured using a spectrophotometric detector at λ = 260 nm.

Analysis of the chromatograms captured for the free protein and its mixture with DNA confirms that in the absence of a crosslinker, there is no non-covalent interaction between these molecules (See Figure S1a). It is evidenced by the unchanged retention time of the free protein and the absence of additional signals indicating the presence of protein-DNA associates. This observation supports the postulated mechanism of covalent coupling with the participation of protein amino groups as the main reaction responsible for forming protein-DNA conjugates. To confirm the specificity of amino-coupling and to verify the relevance of the influence of the possible side reaction of active NHS esters coupling to primary amine groups within nucleobases (thus crosslinking DNA strands at the activation step), the effect of the amine-reactive crosslinker on the ion chromatogram of the ligand strand DNA was also checked. Such a reaction occurring with noticeable efficiency could significantly disrupt the stoichiometry and homogeneity of the DNA-protein conjugates. As can be seen in Figure S1b, there is no noticeable change in the character of the peak from the unmodified DNA sequence after the incubation with the crosslinker compared to the signal for the same sequence without the crosslinker addition. It demonstrates the absence of signs of reaction between the active NHS ester

and -NH2 groups of DNA nucleobases. The selectivity of the activation reaction can be explained by the significantly attenuated basicity of exocyclic amines compared to aliphatic ones [1,2]. Therefore, the DNA nucleobases show poor reactivity towards NHS esters at room temperature [3,4]. Intensities of signals corresponding to the conjugate fractions of various proteins on the chromatograms (*anti*-SARS CoV-2 nucleoprotein in Figure 1a and *anti*-hCRP antibody in Figure 1b, respectively) were compared. It leads to the conclusion that the efficiency of amine-coupling significantly depends on the type of protein. In the case of nucleocapsid antigen, the average conjugation yield for three independent batches (calculated in relation to the total amount of DNA used in the reaction) amounted to 57.6 % ± 8.3 %. In contrast, the efficiency was significantly lower for antibodies, reaching 12.9 % ± 2.0 %. Both calculations were based on the areas of the obtained signals apparent in the chromatograms. Their complex structure may explain the lower coupling efficiency with antibodies. Antibody as a large glycoprotein presumably shows worse reactive amino group availability than recombinant protein without additional modifications. In addition, the lower pI value of the antibody compared to the SARS CoV-2 nucleoprotein (5.8–7.5 vs. 10.2) impedes the interaction of polyanionic DNA chains with the antibody in the conjugation medium due to more negative charge over a wide pH range and thus increased repulsion of polyanionic DNA tags [5,6]. Noteworthy, to increase the efficiency of conjugation with respect to the protein (which is usually a more expensive and less accessible component), a molar excess of DNA is used. This approach further supports the importance of procedures for the purification and fractionation of protein-DNA conjugates due to the need to remove excess free ligands from the reaction mixture.

Figure S2

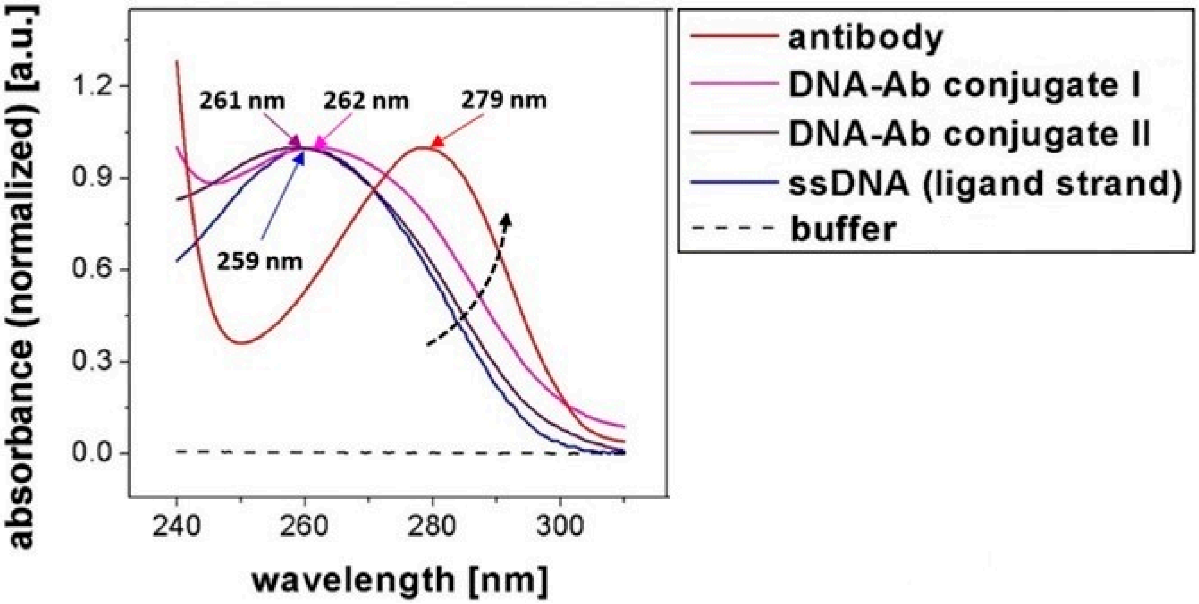

Figure S2. Normalized absorption spectra of selected fractions of post-conjugation mixture as a result of DNA-*anti-h*CRP antibody conjugate separation.

Analysis of UV absorption spectra indicates that DNA-protein conjugates exhibit characteristics of both components. It is evidenced by the observed bathochromic shift relative to free DNA (λ_{max}: 259 nm -> 261 nm for DNA-rich fraction conjugate II and λ_{max}: 259 nm -> 262 nm – for a fraction of 1:1 stoichiometry conjugate I), which can be attributed to the increasing proportion of protein (λ_{max} = 279 nm) in the conjugate. Due to differences in molar extinction, the proximity of the characteristic absorption bands of proteins and DNA, and the low concentration of the tested conjugates, it is impossible to observe independent bands of the two components. Therefore, UV absorption spectra analysis cannot be the only method to validate the efficiency of DNA conjugation with proteins. However, UV absorption analysis can be treated as an auxiliary method to ion chromatography, which does not cause destruction and loss of the sample and allows for the quantitative determination of the conjugate concentration. Despite the limitations mentioned above, UV absorption spectroscopy appears to be an attractive and rapid method for the characterization and quality control of the previously purified DNA-protein conjugates.

Figure S3

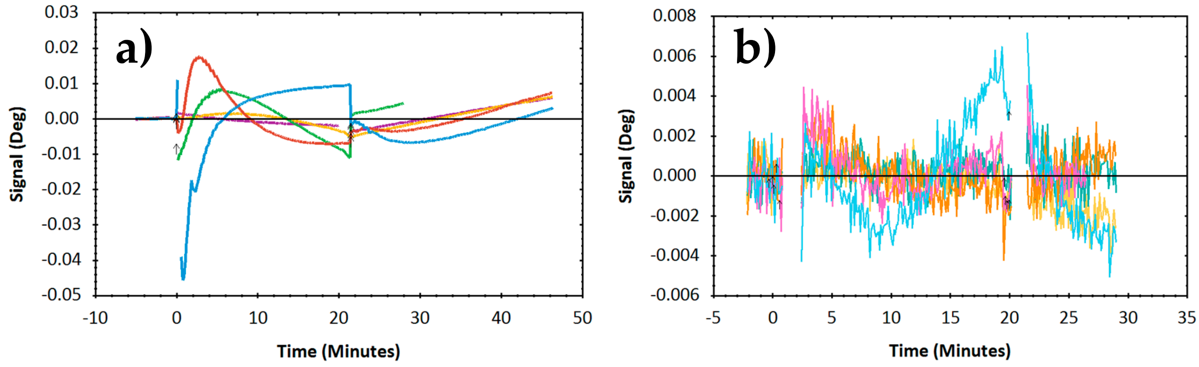

Figure S3. Residuals between fitted curves and experimental data for interactions of *DNA probe* with free *ligand strand* (a) and DNA-*anti*-CRP antibody conjugate (b).

Table S1

Table S1. Detailed statistics data characterizing the used kinetics binding model.

Parameter	Free DNA	DNA-protein conjugate
Fitting model	"one-to-one"	"one-to-one"
Concentration range	17.3 nM – 10.8 μM	0.97 nM – 15.5 nM
B_{max} [mdeg]	140 (± $1.72{\cdot}10^{-1}$)	40.0 (± $8.08{\cdot}10^{-3}$)
k_a [$M^{-1}{\cdot}s^{-1}$]	$1.51{\cdot}10^{5}$ (± $2.35{\cdot}10^{3}$)	$1.13{\cdot}10^{5}$ (± $1.35{\cdot}10^{1}$)
k_d [s^{-1}]	$2.95{\cdot}10^{-4}$ (± $1.56{\cdot}10^{-6}$)	$5.89{\cdot}10^{-4}$ (± $1.97{\cdot}10^{-7}$)
k_D [M]	$1.95{\cdot}10^{-9}$ (± $4.07{\cdot}10^{-11}$)	$5.19{\cdot}10^{-9}$ (± $2.58{\cdot}10^{-12}$)
Chi^2	0.00	0.00

Table S2

Table S2. A comparison of the different types of oligonucleotide probes examined within this study.

Parameter	DNA	ZNA	PNA
Immobilization efficiency (normalized)	100% (15nb – reference value) 100.0% (48nb)	19.1%	24.5%
Hybridization efficiency (normalized)	46.2% (15nb) 46.8 (48nb)	100% (reference value)	0.1%
Surface charge (zeta potential)	-38.2 mV (+/- 1.7mV)	+ 47.8 mV (+/- 3.7mV)	not measured
Probe synthesis cost	low	moderate	high

References:

1. Voigt, N.V.; Tørring, T.; Rotaru, A.; Jacobsen, M.F.; Ravnsbæk, J.B.; Subramani, R.; Mamdouh, W.; Kjems, J.; Mokhir, A.; Besenbacher, F.; et al. Single-Molecule Chemical Reactions on DNA Origami. *Nat Nanotechnol* **2010**, *5*, 200–203, doi:10.1038/NNANO.2010.5.

2. Gothelf, K.V. Chemical Modifications and Reactions in DNA Nanostructures. *MRS Bull* **2017**, *42*, 897–903, doi:10.1557/mrs.2017.276.

3. Verdolino, V.; Cammi, R.; Munk, B.H.; Schlegel, H.B. Calculation of PKa Values of Nucleobases and the Guanine Oxidation Products Guanidinohydantoin and Spiroiminodihydantoin Using Density Functional Theory and a Polarizable Continuum Model. *Journal of Physical Chemistry B* **2008**, *112*, 16860–16873, doi:10.1021/jp8068877.

4. Bryantsev, V.S.; Diallo, M.S.; Goddard III, W.A. PKa Calculations of Aliphatic Amines, Diamines, and Aminoamides via Density Functional Theory with a Poisson-Boltzmann Continuum Solvent Model. *Journal of Physical Chemistry A* **2007**, *111*, 4422–4430, doi:10.1021/jp071040t.

5. Frato, K.E.; Schleif, R.F. A DNA-Assisted Binding Assay for Weak Protein-Protein Interactions. *J Mol Biol* **2009**, *394*, 805–814, doi:10.1016/j.jmb.2009.09.064.

6. Tang, Y.; Cain, P.; Anguiano, V.; Shih, J.J.; Chai, Q.; Feng, Y. Impact of IgG Subclass on Molecular Properties of Monoclonal Antibodies. *MAbs* **2021**, *13*, doi:10.1080/19420862.2021.1993768.
